# Comparison of endovascular coiling and surgical clipping for the treatment of intracranial aneurysms: A prospective study

**Published:** 2015-01-05

**Authors:** Zeinab Taheri, Mohammad Hosein Harirchian, Hosein Ghanaati, Alireza Khoshnevisan, Payman Salamati, Mojtaba Miri, Kavous Firouznia, Mina Saeednejad, Madjid Shakiba, Vafa Rahimi-Movaghar

**Affiliations:** 1 Department of Neurology, Iranian Center of Neurological Research, Tehran University of Medical Sciences, Tehran, Iran; 2 Department of Radiology, Advanced Diagnostic and Interventional Radiology Research Center, Tehran University of Medical Sciences, Tehran, Iran; 3 Department of Neurosurgery, Shariati Hospital, Tehran University of Medical Sciences, Tehran, Iran; 4 Department of Community Medicine, Sina Trauma and Surgery Research Center, Tehran University of Medical Sciences, Tehran, Iran; 5 Department of Neurosurgery, Imam Khomeini Hospital, Tehran University of Medical Sciences, Tehran, Iran; 6 Department of Radiology, Islamic Azad University, Tehran Medical Branch, Tehran, Iran; 7 Department of Radiology, Sina Trauma and Surgery Research Center, Tehran University of Medical Sciences, Tehran, Iran; 8 Department of Neurosurgery, Sina Trauma and Surgery Research Center, Tehran University of Medical Sciences, Tehran, Iran

**Keywords:** Aneurysm, Intracranial, Therapy, Assessment, Outcome

## Abstract

**Background: **Management of intracranial aneurysms has made debates about the best treatment modality in recent years. The aim of this study was to compare the interventional outcomes between two groups of patients, one treated with endovascular coiling and the other treated with surgical clipping.

**Methods:** This prospective study included 48 patients with intracranial aneurysms who underwent endovascular coiling (27 patients) or surgical clipping (21 patients) from July 2011 to August 2013. A neurologist examined patients in admission and followed them by phone call 1-year after intervention.

**Results: **Mean modified Rankin Scale (MRS) score at the time of admission in endovascular group was 2.86 ± 0.974 whereas it was 3.81 ± 1.078 in surgical clipping group (P = 0.0040). Focal neurologic signs were higher in clipping during procedures (P = 0.0310). Of 37 patients who followed up for a year, 19 were in endovascular group and 18 in surgical clipping group. At 1 year follow-up, MRS improvement was statistically significant in coiling group (P = 0.0090), but not in clipping group (P = 0.8750). Mean difference of MRS score at the time of admission and at one year later, was 0.947 ± 1.224 in endovascular group and 0.111 ± 2.083 in surgical group (P = 0.3000).

**Conclusion: **There was no statistically significant difference at 1 year outcome between two groups. We recommend further interventional studies with larger sample sizes for better evaluation of the modalities.

## Introduction

Aneurysmal subarachnoid hemorrhage (aSAH) is a disastrous and fatal medical emergency requiring immediate intervention as approximately 12% of patients die before receiving medical supports, 33% within 48 h and 50% within 30 days of aSAH and 50% of survivors suffer from permanent disability and dependency.^1^ Endovascular coiling has increasingly become an alternative procedure for surgical clipping in both ruptured and unruptured aneurysms in last decades.^2^^,^^3^ According to the study of Hwang et al., the majority of ruptured and unruptured aneurysms were coiled in US in 2002-2008.^3^ However, there are considerable risks and complications such as thromboembolism, aneurysm rupture, patent artery occlusion, coil migration and vasospasm in endovascular therapy.^4^ Both modalities have advantages and disadvantages which make them as complementary rather than competitive.^3^ The most prominent advantage of surgical clipping is long term durability which is controversial in endovascular coiling. Long-term follow-up performed by intracranial subarachnoid aneurysm trial (ISAT) indicated that coiling had higher risk of rebleeding than clipping.^4^^-^^6^ The disadvantage of surgical clipping is the fact that it requires open surgery which is accompanied with more morbidity in elderly patients.^7^ Hence, durability of endovascular coiling is not a major concern in this group of patients.^6^ The advantages of endovascular coiling are less invasiveness, easy access to vertebrobasilar system and multiple aneurysms in distant areas.^3^ However, coiling is not useful in all aneurysms as it cannot remove intracerebral hemorrhage or mass effect of giant aneurysms.^8^ In addition, the treatment modality differs significantly in ruptured and unruptured aneurysms.^9^ Although there are numerous studies comparing surgical clipping and endovascular coiling, there is no study investigating outcomes of surgical clipping and endovascular coiling in our country. Our aim was to evaluate surgical clipping and endovascular coiling outcomes by comparing modified Rankin Scale (MRS) before and 1-year after procedure in both groups of patients.

## Materials and Methods

We conducted a descriptive prospective study, including 49 consecutive patients with intracranial aneurysms who underwent endovascular coiling in Imaging Medical Center of Imam Khomeini Hospital and surgical clipping in Neurosurgery Department of Shariati Hospital in Tehran, Iran from July 2011 to August 2013. 

The study included all patients with intracranial aneurysms who were ruptured or unruptured. Patients with mycotic, metastatic, atherosclerotic and dissecting aneurysms, multiple aneurysm (more than 2) and giant aneurysms (more than 25 mm in size) were excluded. 

An independent neurologist examined all the patients at the time of admission and followed them by phone call 1 year after intervention and scored them according to MRS. The scale runs from 0 to 6, running from perfect health without symptoms to death (0- No symptoms; 1- No significant disability. Able to carry out all usual activities, despite some symptoms; 2- Slight disability. Able to look after own affairs without assistance, but unable to carry out all previous activities; 3- Moderate disability. Requires some help, but able to walk unassisted; 4- Moderately severe disability. Unable to attend to own bodily needs without assistance, and unable to walk unassisted; 5- Severe disability. Requires constant nursing care and attention, bedridden, incontinent; 6- Dead.

The diagnoses were confirmed using computed tomography (CT)-scanning and/or magnetic resonance imaging (MRI) and/or angiography. Decision on treatment protocol was made based on patients’ conditions and physician interest. 

All the patients were examined first by neurologist or neurosurgeons and they decided if they want to refer the patient to interventional radiology department for coiling or referred to neurosurgeons for clipping. 

There was a check list filled for all patients at the time of admission including demography, risk factors, MRS before and 1 year after intervention, CT scan, MRI data and procedural information. 

If there were two aneurysms in opposite hemispheres, there would be a particular check list for each one. The research was concordance with ethical consideration of Tehran University of Medical Sciences. 

Extracted data were analyzed with SPSS (version 16, SPSS Inc., Chicago, IL, USA). Chi-square, fisher-exact, independent-t, paired-t and Mann–Whitney U-tests were used and were considered as statistically significant at P < 0.050.

## Results

Twenty-seven patients underwent endovascular coiling and 21 patients underwent surgical clipping. There were 17 females (63%) in coiling group and 13 females (61.9%) in clipping group. Mean age of patients allocated to surgical treatment was 53.7 ± 13.0 and mean age of patients allocated to endovascular therapy was 51.2 ± 11.9 (P = 0.5080). Of 24 patients with history of hypertension, 14 patients (66.7%) were in surgical group and 10 patients (37%) were in coiling group (P = 0.0420). Other risk factors are featured in table 1.

**Table 1:** Comparison of risk factors among two groups

**Risk factors**	**Surgical Clipping [n (%)]**	**Interventional coiling [n (%)]**	**P**
Hyperlipidemia	8 (38.1)	3 (11.1)	0.0270
Smoking	3(14.3)	5 (18.5)	0.9900
Cerebro vascular disease	0	2 (7.4)	0.5000
Polycystic kidney disease	0	1(3.7)	0.9900
Family history of Cerebrovascular diseases	0	1(3.7)	0.9900
Hypertension	14 (66.7)	10 (37)	0.0420

**Table 2:** Anatomical distribution of aneurysms

**Anterior circulation**	**Coiling [n (%)]**	**Clipping [n (%)]**
ICA	13 (56.6)	1 (4.8)
P Com A	1 (4.3)	1 (4.8)
ACA	0	3 (14.3)
A2	0	1 (4.8)
A Com A	3 (13)	5 (23.8)
MCA	6 (26.1)	6 (28.6)
AComA and MCA	0	1 (4.8)
ACA and MCA	0	2 (9.5)
ACA and AComA	0	1 (4.8)
Posterior circulation		
Basilar artery tip	2 (50)	2 (50.0)
Basilar artery trunk	1 (25)	1 (25.0)
Posterior cerebral artery	1 (25)	1 (25.0)

ICA: Internal carotid artery;

ACA: Anterior cerebral artery;

MCA: Middle cerebral artery

P Com A: Posterior communicating artery;

A Com A: Anterior communicating artery

Of 28 patients who presented with ruptured aneurysms at the time of admission, 17 (81%) were in the surgical group and 11 (41%) were in coiling group (P = 0.0050).

Symptoms and signs of the patients at the time of admission were hypertension in 12 (25%), vertigo in 11 (22.9%), severe headache in 27 (58.7%). Four patients (8.7%) were asymptomatic.

Forty-four (91.7%) aneurysms were located in anterior circulation, and 4 (8.3%) were in posterior circulation. The arterial distribution of aneurysms is shown in table 2. Five aneurysms (10.6%) were smaller than 4 mm, 17 (36.2%) were 4-10 mm and 25 (53.2%) were > 10 mm. The detailed distributions of the aneurysms according to their size in two groups have been mentioned in table 3. The percentage of large aneurysms was significantly higher in coiling group. Eighteen aneurysms (40.9%) had wide neck (neck diameter > 4 mm) and 26 aneurysms (59.1%) had narrow neck^8^ 91.7% of aneurysms were saccular (Table 3).

The frequencies of complications during treatment among two groups have been mentioned in table 4. The frequency of focal neurologic signs was significantly higher in the clipping group.

Patients with MRS score of 1, 2 and 3 at the time of admission were 14 (29.2%) in each one. There were 4 (8.3%) persons with MRS score of 4 and 2 (4.2%) with MRS score of 5. There was no patient with MRS score of 0 in each group.

Mean MRS score at the time of admission in the endovascular group was 2.86 ± 0.974 while this figure was 3.81 ± 1.078 in the surgical group (P = 0.0040). Of 37 patients with 1 year follow-up, 19 were in the endovascular group and 18 in the surgical group. Mean MRS score of patients 1 year after procedure was 1.89 ± 0.809 and 3.67 ± 2.223 in the endovascular group and surgical group, respectively (P = 0.0100). MRS improvement is statistically significant in coiling group (P = 0.0090), but not in clipping group (P = 0.8750).

Mean difference of MRS score at the time of admission and 1 year later, was 0.947 ± 1.224 in the endovascular group and 0.111 ± 2.083 in the surgical group (P = 0.3000) (Table 5).

**Table 3:** Comparison of the anatomical properties of aneurysms between two groups

**Procedure**	**Neck size [n (%)]**			**Shape [n (%)]**				**Aneurysm size [n (%)]**			
	**Wide**	**Narrow**	**P **	**Saccular**	**Fusiform**	**Other**	**P **	**Micro**	**Small 4-10 mm**	**Large > 10 mm**	**P **
Coiling	13 (50.0)	13 (50.0)	0.1400	24 (88.9)	2 (7.4)	1 (3.9)	0.9900	2 (7.4)	6 (22.2)	19 (70.4)	0.0230
Clipping	5 (27.8)	13 (72.2)		20 (95.2)	1 (4.8)	0		3 (15.0)	11 (55.0)	6 (30.0)	

**Table 4:** Complications during procedure

**Complication**	**Coiling [n (%)]**	**Clipping [n (%)]**	**P **
Rupture	1 (3.8)	0 (0)	1.0000
Brain infarction	1 (3.7)	3 (14.3)	0.3060
Focal neurological signs	2 (7.4)	7 (33.3)	0.0310
Vision disturbance	0	2 (10)	0.1840
Death	0	1 (4.8)	0.4470

**Table 5:** Mean modified Rankin Scale (MRS) scores before and at 1 year after the treatment and their difference in two groups

**Procedure**	**Mean MRS at the time of admission**	**Mean MRS at 1 year follow-up**	**P **	**Difference**
Coiling	2.86 ± 0.974	1.89 ± 0.809	0.0090	0.947 ± 1.224
Clipping	3.81 ± 1.078	3.67 ± 2.223	0.8750	0.111 ± 2.083
P	0.0040	0.0120		0.3000

MRS: Modified Rankin scale

The distribution of different MRS scores at the time of admission and 1 year post-intervention in two groups are shown at table 6. Regarding the change of MRS in patients, there were 2 cases (10.5%) of deterioration of MRS in coiling and 7 cases (38.9%) of deterioration in clipping group. In addition, the frequency of MRS improvement was higher in coiling group, but there was not statistically significant difference between two groups (Table 7).

Hydrocephalus occurred in 4 patients at 1 year follow-up all in surgical clipping (P = 0.0350). There was no statistically difference in other complications at 1 year follow-up in two groups. (seizure seen in one patient in clipping group, infection seen in one patient in clipping group and pulmonary complication seen in one patient in the clipping group).

## Discussion

This study has been done to compare the surgical clipping with endovascular coiling in treatment of brain aneurysms in terms of risk factors, preprocedural clinical findings of the patients, anatomical properties of the aneurysms, procedural complications and 6 months follow-up disability status based on MRS.

The endovascular coiling has been introduced as the brain aneurysm treatment since 1990s and approved as a relatively minimal invasive method.^4^ Few studies have focused on the comparison of the coiling and clipping in the treatment of disease.

By daily advancing in non-invasive imaging techniques such as CT and MR angiography, there are more unruptured intracranial aneurysms (UIA) incidentally detected, which management strategy remains controversial. Several studies revealed that the risk of rupture for UIAs is estimated 1% per year for aneurysms 7-10 mm in diameters.^10^^,^^11^ The study of Ishibashi et al. showed that a previous history of SAH, the posterior circulation location and large size were significantly predictors of aneurysm rupture.^12^ ISAT showed that endovascular coiling is the treatment of choice for ruptured intracranial aneurysms rather than neurosurgical clipping on patients suitable for either treatment, although the difference in morbidity and rate of independency decreases over time.^2^^,^^13^

In this paper, we assessed two groups of ruptured or non-ruptured brain aneurysms treated with coiling and clipping. The patients were followed up for 1 year and their disability was assessed before treatment and after this period. Our study was not randomized thus the baseline situation of the patients was not similar between two groups. Specifically, the clipping group had more ruptured aneurysms in comparison to coiling patients. This could be due to the initial situations of the patients entered the treatment facilities. Naturally, most of the patients with ruptured aneurysms referred to the hospital emergencies and then they were referred to the neurosurgery services. In this situation, they are more probable to be operated due to their emergent situation.

**Table 6:** Distribution of modified Rankin Scale (MRS) scores in two groups before and at 1 year after the treatment

**MRS score**	**Coiling** ** [n (%)]**		**Clipping ** **[n (%)]**	
	**At the time of admission **	**At 1 year follow-up **	**At the time of admission **	**At 1 year follow-up **
0	0	6 (31.6)	0	4 (22.2)
1	12 (44.4)	10 (51.6)	2 (9.5)	2 (11.1)
2	8 (29.6)	2 (10.5)	6 (28.6)	4 (22.2)
3	5 (18.5)	1 (5.3)	9 (42.9)	2 (11.1)
4	2 (7.4)	0	2 (9.5)	2 (11.1)
5	0	0	2 (9.5)	0
6	0	0	0	4 (22.2)

MRS: Modified Rankin Scale

**Table 7:** Trend of modified Rankin Scale (MRS) among two groups before and after the treatment

	**Coiling [n (%)]**	**Clipping [n (%)]**	**P**
Deteriorated	2 (10.5)	7 (38.9)	0.1900
Unchanged	4 (21.1)	2 (11.1)	
Improved	13 (68.4)	9 (50.0)	

MRS: Modified Rankin Scale

In addition, the patients underwent coiling had greater sized aneurysms in comparison to clipping group and the frequency of internal carotid artery aneurysms was higher than clipping group. These could be due to the fact that these aneurysms are more difficult to be operated by clipping method.

When we consider the MRS, scores in two groups, the baseline score of clipping group was worse. This could be because more patients in clipping group experienced rupture and SAH before the treatment. Worse MRS in the clipping group at the time of the procedure could be the cause of worse MRS at 1 year follow-up. Although the MRS difference of pre- and post-intervention in coiling group was greater than clipping group, this was not statistically significant between two groups.

Regarding the comparison of two treatment methods, some studies, and systematic reviews and meta-analyses have been published. These reviews are separately published on the ruptured and unruptured aneurysms. One of the most famous trials was ISAT. Up to now, multiple periodical reports have been published on the patients entered in this international follow-up trial. They have reported the comparison of the safety and efficacy of endovascular coiling versus neurosurgical clipping in patients with a ruptured intracranial saccular aneurysm. In addition, there are a few reports and systematic reviews on the unruptured brain aneurysms.

Brinjikji et al. in a study on medical records of 64,043 patients with unruptured aneurysms found coiling treatment increased from 20% in 2001 to 63% in 2008. In addition, they showed the percentage of patients discharged to long-term facilities were 14.0% (4184/29,918) in surgical clipping while it was 4.9% (1655/34,125) in coiling patients (P < 0.0001). In addition, patients underwent clipping had a higher mortality rate (1.2% vs. 0.6%, P < 0.0001). Between 2001 and 2008, the total percentage of adverse events after treatment dropped from 14.8% to 7.6%. They concluded that in unruptured aneurysms, coiling in comparison to clipping is associated with lower periprocedural morbidity and mortality in the time period of 2001-2008 in USA.^14^

Hwang et al. performed a systematic review comparing endovascular coiling versus neurosurgical clipping on patients with UIA performed according to 24 included studies (n = 31,865). Their outcome measures were glasgow outcome scale (GOS) and MRS. They showed clipping is significantly associated with higher disability based on GOS [odds ratio (OR) = 2.38; 95% confidence interval (CI) = 1.33-4.26] and MRS (OR = 2.83; 95% CI = 1.42-5.63). Comparison of complications showed worse profile for clipping in neurological and cardiac complications: 1.94 (95% CI = 1.09-3.47) and 2.51 (95% CI = 1.15-5.50), respectively. Furthermore, in clipping, short-term (≤ 6 months) disability for GOS was significantly greater (OR, 2.72; 95% CI = 1.16-6.34), but not in the long term (> 6 m) GOS (OR, 2.12; 95% CI = 0.93-4.84). They concluded that considering disability and complication in a short term, coiling is a better procedure for patients with unruptured aneurysm. However, the level of evidence for this finding is low due to the limitations of included studies; further investigations are needed for a stronger conclusion.^3^

The results of these two studies are similar to ours as the disability in our study seems to be smaller after coiling in terms of MRS. Yet, in our study, the MRS change was not different between two groups. We should consider that our patients had both ruptured and unruptured aneurysms. This makes a difference between these studies and ours as the mentioned studies only recruited unruptured aneurysms.

Li et al in a systematic review on patients with ruptured intracranial aneurysms between 1999 and 2012 included four randomized controlled trials and 23 observational studies showed according to randomized controlled trials results, coiling reduces the 1 year unfavorable outcome rate (OR = 1.48; 95% CI = 1.24-1.76), but no statistical difference in nonrandomized controlled trials (OR = 1.11; 95% CI = 0.96-1.28). Compared with patients with poor preoperative condition, good preoperative grade patients treated with coiling showed better outcomes (OR = 1.51; 95% CI = 1.24-1.84 vs. OR, 0.88; 95% CI = 0.56-1.38). Incidence of rebleeding was higher after coiling (OR = 0.43; 95% CI = 0.28-0.66), while complete occlusion rate of clipping was better (OR = 2.43; 95% CI = 1.88-–3.13). The 1 year mortality rate was similar. Vasospasm was more common after clipping whereas the ischemic infarct shunt-related hydrocephalus and procedural complication rates did not show any difference between techniques. They concluded coiling is associated with a higher risk of rebleeding, but yields a better clinical outcome especially in those patients with a good preoperative status.^15^

Hoh et al. in a study on 515 patients with aneurysmal SAH treated with coiling (n = 79), clipping (n = 413) or clipping with craniotomy for any reason (n = 23) considered vasospasm has poor outcome (according to modified Rankin score of 3-6) and in-hospital mortality. In their retrospective single center and nonrandomized study, clipping had a better outcome than coiling among good situation patients without any effect on vasospasm.^16^

Taha et al. in a retrospective study on 133 patients hold 168 aneurysm (ruptured or unruptured) showed better follow-up angiographic results in clipping (total occlusion of 81.4% vs. 57.5%). In SAH patients, the frequency of vasospasm after angiography was 17.4% in coiling and 45.4% in clipping. In SAH patients, excellent outcome for coiling and clipping groups was seen in 62% and 44%, respectively. However, in unruptured patients, this profile was 93% vs. 81%, respectively. They concluded in ruptured and unruptured cerebral aneurysms, coiling is a safe alternative for clipping.^17^

One of the advantages of coiling is that it could be done on patients with poor condition. Weir et al. in a study on the SAH patients with Hunt and Hess grade of 4 or 5 showed despite poor medical condition and a high frequency of vasospasm during treatment these patients can undergo successful coil embolization, but morbidity and mortality are still high. These findings compare favorably with similar patients treated aggressively in surgical series.^18^

Molyneux et al. in a report of 2143 patients recruited in ISAT in Europe centers reported 1 year outcomes for 1063 patients allocated to endovascular treatment, and 1055 patients allocated to neurosurgical treatment. Among endovascular treatment patients, 250 (23·5%) were dead or dependent at 1 year, compared with 326 (30·9%) of patients underwent neurosurgery. The absolute risk reduction was 7·4% (95% CI = 3·6-11·2, P = 0·0001). The early survival advantage was maintained for up to 7 years and was significant (log-rank P = 0·0300). The risk of epilepsy was substantially lower in endovascular treated patients, while the risk of late rebleeding was higher.^19^

Renowden et al. in a study on SAH patients treated with coil embolization reported their 10-year experience. They showed failed technique in 25 patients among 717. Rupture complicated Thirty-seven procedures (4.7%) resulted in 10 permanent disability or dead (1.3%). Thromboembolic events were seen in 35 procedures (4.5%) resulting in 8 permanent disability or dead. Six procedures were complicated by dissection. Overall morbidity or mortality was 2.9%. Sixteen patients experienced another subarachnoid hemorrhage (2.3%) resulting in 12 death. At 6 months, 580 patients (82%) were independent, and 130 patients (18%) were disabled or dead. They concluded that coiling is a feasible treatment with a small mortality and permanent morbidity risk and without a high risk of rebleeding. They showed majority of patients recovered independently.^20^ Our results are comparable with the results of this study.

Sturiale et al. in a systematic review on endovascular treatment in elderly patients recruited 21 studies reporting totally 1511 patients. Long-term aneurysm occlusion rate was 79% (95% CI = 70-85%). 4% experienced perioperative stroke (95% CI = 3-6%) (similar finding among ruptured and unruptured aneurysms). Rupture during procedure occurred in 1% and 4% of patients with unruptured and ruptured aneurysms, respectively. Perioperative mortality rate was greater in SAH patients (23% vs. 1%; P < 0.0100). Good clinical outcome at 1 year were 93% and 66% in patients with unruptured and ruptured aneurysms, respectively. They concluded that coiling in elder patients has a long-term occlusion rate, but regarding the morbidity and mortality in this treatment, careful patient selection is recommended especially in unruptured aneurysms.^21^

In a study done on the ISAT database for comparing recurrence of SAH, dependence and standardized mortality ratios between coiling and clipping, it was shown an increased small risk of recurrence in coiling while 5 years risk of death was significantly higher in clipping.^22^

One important point in large scale comparison of two procedures is the cost analysis. Of course, the total final cost also depends to the initial situation of the patients regarding the aneurysm rupture. The patients could be discharged to home, short term facilities, long term facilities or could be dead. These situations are associated with different costs. The greater cost has been shown in patients discharged to long term facilities.^23^ In addition, the hospital stay after the procedure is very important in the total cost of procedure. This cost differs between different countries because it is related to the fundamental economic system of the country. In one study performed in the USA in 2008, it was determined costs of patients discharged to home or short term facilities was higher in patients under 65 who underwent clipping (in comparison to coiling) and this pattern was also seen in patients greater than 65 years old who discharged to home.^23^ However, in the mentioned study, considering all patients, the total cost in two groups did not show statistical difference.^23^ There is not any comprehensive cost analysis study in our country comparing the clipping versus coiling, but it seems regardless of physician payment, the most part of costs in clipping treatment relates to hospital stay while in coiling, it relates to the coil preparation. Hoh et al in a study of NIS in 2002-2006 found that the clipping patients experienced significantly higher hospital stay and total hospital costs than coiling among patients with ruptured aneurysms.^24^

Our study had some limitations. Patients were not good samples for comparison because of dissimilarity in ruptured aneurysms percentage, aneurysm size, aneurysm location and MRS at the time of enrollment in the study. Patients were not randomly assigned in each group and moreover, our sample size was small, and it could lead to observed insignificant differences. Hence, the results of this research should be interpreted cautiously.

## Conclusion

There were no statistically significant differences in 1-year outcomes between two groups. We recommend further interventional studies with bigger sample sizes for better evaluation of the modalities.

## Notes

**How to cite this article: **Taheri Z, Harirchian MH, Ghanaati H, Khoshnevisan A, Salamati P, Miri M, et al. Comparison of endovascular coiling and surgical clipping for the treatment of intracranial aneurysms: A prospective study. Iran J Neurol 2015; 14(1): 22-8.
